# SIRT1 Activators as Geroprotective Agents in Brain Aging: Mechanisms and Therapeutic Potential

**DOI:** 10.1007/s12017-026-08923-y

**Published:** 2026-04-04

**Authors:** Ayman Ali Mohammed Alameen, Hayder M. Al-Kuraishy, Ali I. Al-Gareeb, Athanasios Alexiou, Marios Papadakis, Safaa A. Faheem, Gaber El-Saber Batiha

**Affiliations:** 1 Department of Clinical Laboratory Sciences, College of Applied Medical Sciences, Jouf University, P.O. Box 2014, Sakaka, Saudi Arabia; 2 Department of Clinical Pharmacology and Medicine, College of Medicine, Al-Mustansiriya University, Baghdad, Iraq; 3 European Academy of Sciences and Arts, Vienna, Austria; 4 University Centre for Research & Development, Chandigarh University, Chandigarh-Ludhiana Highway, Mohali, Punjab India; 5 Medical Department, Faculty of Life Sciences, University of Thessaly, 3 Panepistimiou Str., Viopolis, 41500 Larissa Greece; 6 Department of Pharmacology and Toxicology, Faculty of Pharmacy, Egyptian Russian University, Cairo-Suez Road, Badr City, Cairo, 11829 Egypt; 7 Department of Pharmacology and Therapeutics, Faculty of Veterinary Medicine, Damanhur University, Damanhur, AlBeheira, 22511 Egypt

**Keywords:** SIRT1, Brain aging, Geroprotectors, Neuroinflammation, Oxidative stress

## Abstract

The brain undergoes profound molecular and structural changes during the aging process, resulting in the development of neurodegeneration, cognitive impairment, and increased vulnerability to chronic diseases. At the cellular level, brain aging is characterized by oxidative damage, genomic instability, and chronic low-grade inflammation known as inflammaging. Central to this process is Sirtuin 1 (SIRT1), a NAD^+^-dependent class III histone deacetylase, known for its regulatory role in chromatin remodeling, oxidative stress responses, mitochondrial biogenesis, and neuroplasticity. Recent research has identified SIRT1 as a molecular target capable of reversing or attenuating several hallmarks of aging, particularly within the central nervous system (CNS). This narrative review critically evaluates the emerging evidence surrounding the geroprotective effects of SIRT1 activators, which exert dual actions, senomorphic and senolytic, via modulation of signaling pathways, thereby reducing neuronal senescence, enhancing autophagy, and mitigating inflammatory responses. The discussion also addresses the region-specific role of SIRT1 across the brain, particularly in the hippocampus and hypothalamus, which are essential for memory, energy homeostasis, and resilience to stress. Additionally, this review explores how SIRT1 depletion during aging contributes to the development of synaptic dysfunction, impaired cognitive function, and susceptibility to neurodegenerative diseases such as Alzheimer’s disease (AD) and Parkinson’s disease (PD). The therapeutic potential of SIRT1 activators is supported by preclinical and early clinical studies, suggesting their value in preventing or delaying brain aging. Thus, SIRT1 could be a promising pharmacological target for age-associated brain disorders, warranting more robust translational studies to validate these findings in humans.

## Background

Aging is a multifactorial process that affects different tissues and organs, including the brain, and is influenced by genetic and environmental factors (Cai et al., 2022). The primary processes related to aging are progressive accumulation of inflammatory molecules, chronic inflammation, and the development of aberrant cellular signaling (Chung et al., 2019). During human life, the cellular system is frequently exposed to external genotoxic factors, such as chemical and physical agents, and internal genotoxic factors, which increase the risk of DNA injury and mutagenesis and dysregulate the neuroprotective silencing information regulator 2-related enzyme 1 (SIRT1) (Rosendahl Huber et al., 2021). SIRT1 is strongly downregulated during brain aging, leading to increased cellular senescence (Sung et al., 2021). Therefore, increasing brain SIRT1 signaling can attenuate the development and progression of brain aging.

On the other hand, geroprotectors, which inhibit aging and extend lifespan, have been increasingly suggested to play a critical role in aging by modulating the SIRT1 signaling pathway (Morsli & Bellantuono, 2021). Notably, SIRT1 activation acts as an anti-aging mechanism by modulating the SIRT1/acetyl-NF-κB signaling axis and stimulating autophagy (Morsli & Bellantuono, 2021). Accordingly, this review highlights the potential protective role of SIRT1 and the geroprotective effects of SIRT1 activators in brain aging. The therapeutic potential of SIRT1 activators is supported by different preclinical and early clinical studies, suggesting their value in preventing or delaying brain aging. Thus, SIRT1 could be a promising pharmacological target for age-associated brain disorders, warranting more robust translational studies to validate these findings in humans. Therefore, this narrative review aims to discuss, explain, and critically evaluate the emerging evidence for SIRT1 activators that modulate various cellular and molecular signaling pathways in brain aging.

## Method and Search Strategy

### Literature Search Strategy

This article is a narrative review summarizing current evidence on the role of SIRT1 in brain aging and its potential therapeutic modulation. A literature search was performed across major scientific databases, including PubMed, Scopus, Embase, Cochrane Library, and CENTRAL, to identify relevant studies published up to January 2026.

The search strategy used combinations of keywords and Medical Subject Headings (MeSH) terms such as “SIRT1,” “brain aging,” “neurodegeneration,” “SIRT1 activators,” and “neuroprotection.” Boolean operators (AND/OR) were applied to refine the search.

Relevant experimental studies (in vitro and in vivo), clinical studies, and mechanistic investigations examining the relationship between SIRT1 signaling and brain aging or neurodegenerative processes were considered. The reference lists of relevant publications were also screened to identify additional articles.

Studies were selected based on their relevance to SIRT1 mechanisms in brain aging, neuroprotection, and associated signaling pathways. The findings from the selected literature were synthesized narratively, with emphasis on mechanistic insights, experimental evidence, and potential therapeutic implications.

## The Physiological role of SIRT1

SIRT1 is an NAD^+^-dependent deacetylase involved in regulating cellular senescence, aging, and other cellular and molecular processes (Table 1). SIRT1 plays a critical role in genomic stability during aging and regulates aging and cellular senescence (Chen et al., 2020). The expression of SIRT1 declines with aging, though increased SIRT1 expression is sufficient to extend lifespan in yeast and mice (Chen et al., 2020). Notably, SIRT1 regulates gene expression by deacetylating acetyl-lysine residues on both histone and non-histone substrates. SIRT1 also interacts with proteins in multiple signaling pathways, thereby influencing a wide range of biological, physiological, and pathological processes (Cui et al., 2022). For instance, SIRT1 suppresses the release of inflammatory mediators by downregulating NF-κB expression, thereby mitigating toxicant-induced inflammatory damage (Cui et al., 2022). Although the mechanism of SIRT1-activating compounds (STACs) remains debated, evidence suggests that specific hydrophobic motifs in SIRT1 substrates, such as peroxisome proliferator-activated receptor gamma co-activator 1 alpha (PGC-1α) and FOXO3a, enhance SIRT1 activation (Hubbard et al., 2013). A single amino acid, Glu230, located in the structured N-terminal domain of SIRT1, is essential for activation by both conventional STAC scaffolds and newer chemically distinct activators. Notably, in primary cells expressing activation-deficient SIRT1, the metabolic effects of STACs are abolished (Hubbard et al., 2013). Furthermore, SIRT1 is involved in aging and longevity by modulating signaling pathways, such as PGC-1α, which promotes mitochondrial biogenesis. SIRT1 is located in the nucleus and can deacetylate transcription factors and histone enzymes, including histone methyltransferases (Yang et al., 2022).

**Fig. 1:**
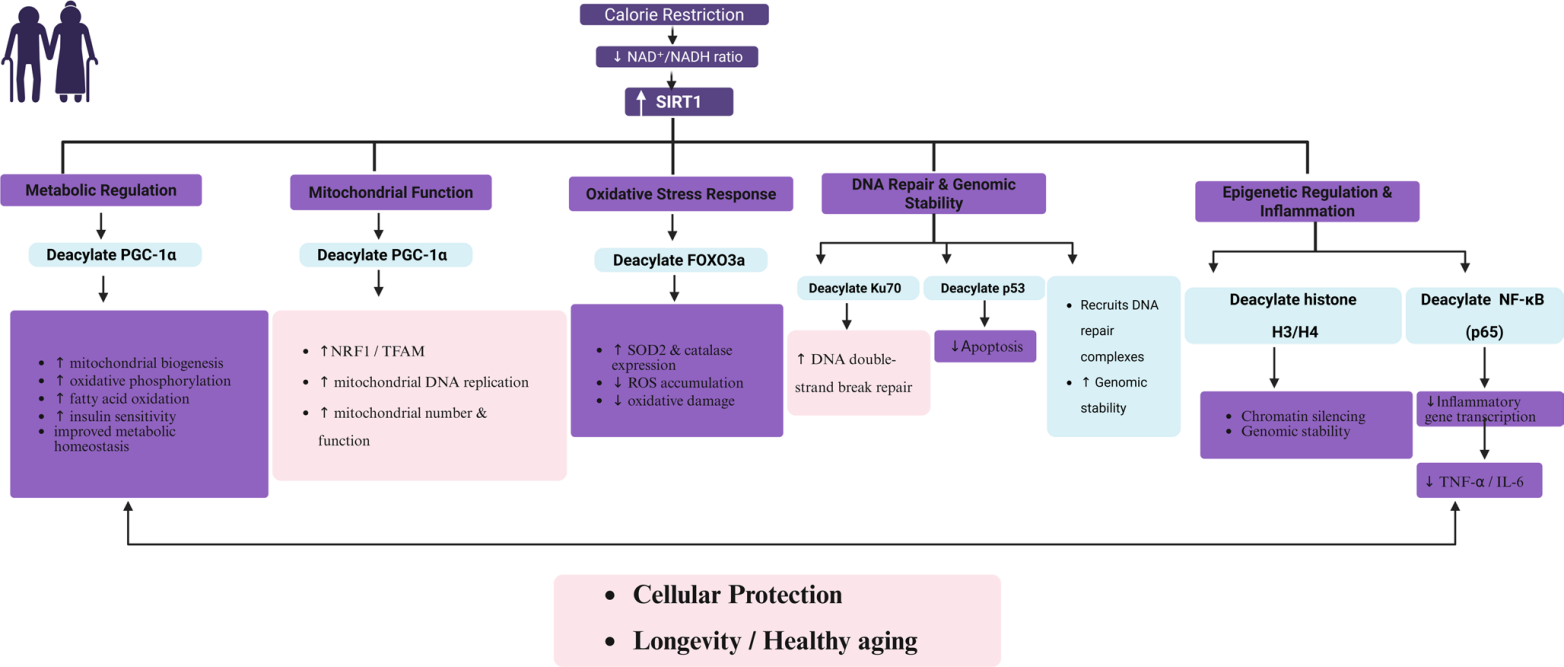
Mechanistic overview of the physiological roles of SIRT1 in cellular homeostasis and longevity. Caloric restriction and metabolic cues increase SIRT1 activity through NAD⁺-dependent signaling. Activated SIRT1 exerts pleiotropic effects via deacetylation of key histone and non-histone substrates. In metabolic regulation and mitochondrial function, SIRT1 deacetylates PGC-1α, promoting activation of the NRF1–TFAM axis, mitochondrial DNA replication, oxidative phosphorylation, fatty acid oxidation, and improved metabolic homeostasis. In the oxidative stress response, SIRT1 deacetylates FOXO3a, enhancing transcription of antioxidant enzymes such as superoxide dismutase 2 (SOD2) and catalase, thereby reducing reactive oxygen species (ROS) accumulation and oxidative damage. For DNA repair and genomic stability, SIRT1 modulates Ku70 and p53 signaling, enhances DNA double-strand break repair, recruits DNA repair complexes, and regulates apoptosis. In epigenetic regulation and inflammation control, SIRT1 deacetylates histones (H3/H4), promoting chromatin silencing and genomic stability, and suppresses NF-κB (p65)-mediated transcription of pro-inflammatory genes, leading to reduced expression of cytokines such as TNF-α and IL-6. Collectively, these integrated mechanisms contribute to cellular protection, reduced inflammation and apoptosis, enhanced mitochondrial integrity, and promotion of healthy aging and longevity

Interestingly, SIRT1 promotes apoptotic cell death and improves protein stability by regulating insulin-like growth factor 1 (IGF-1) and PI3K (Batiha et al., 2023; Xu et al., 2020; Yang et al., 2022). Importantly, caloric restriction is regarded as a key mechanistic pathway for SIRT1 activation, leading to PGC-1α and histone activation, and to the suppression of p53 and NF-κB (Batiha et al., 2023; Xu et al., 2020; Yang et al., 2022). These integrated mechanisms are summarized in Fig. 1. Although there is considerable interest in enhancing SIRT1 catalytic activity, its regulation at the protein level remains poorly understood. Evidence indicates that macroautophagy, a catabolic pathway involving autophagosomes and lysosomes, drives the downregulation of mammalian SIRT1 during cellular senescence and aging (Xu et al., 2020). In senescent cells, nuclear SIRT1 is an autophagy substrate and degraded through cytoplasmic autophagosome-lysosome activity mediated by the autophagy protein LC3. This autophagy-lysosome pathway has been shown to contribute to the decline of SIRT1 in aging tissues (Xu et al., 2020). Therefore, modulating autophagy may represent a potential approach to stabilize SIRT1 and support healthy aging. Such effects could reduce inflammation and apoptosis, enhance genomic stability and mitochondrial biogenesis, and ultimately promote longevity.

Also, SIRT1 increases antioxidant expression, resulting in a significant reduction in oxidative stress and inhibition of inflammatory responses in aging (Al-Kuraishy et al., 2023; Yang et al., 2022). SIRT1 affects numerous biological processes by deacetylating diverse proteins involved in inflammation. Evidence suggests that SIRT1 is downregulated during the acute inflammatory response and related diseases, both in vivo and in vitro (Yang et al., 2022; Zhang et al., 2010). Conversely, while SIRT1 significantly reduced the inflammatory reactions to IL-1β and TNF-α in human dermal microvascular endothelial cells (HDMEC), no significant change in SIRT1 expression was observed in psoriatic HDMEC treated with IL-1β, IFN-γ, and IL-17 (Orecchia et al., 2011). Interestingly, both NF-κB and oxidative stress, which are elevated with aging, inhibit SIRT1 expression (Cui et al., 2022). However, optimal levels of ROS and nitrogen oxidative species (NOS) are necessary for cellular molecular signaling (Ivanova & Lyublinskaya, 2021), and excessive use of antioxidants can induce redox imbalance, leading to a harmful condition called antioxidant stress (Ge et al., 2022). Therefore, SIRT1, which regulates oxidative stress and antioxidant balance, appears to be a more appropriate signaling pathway for preventing aging and is considered a geroprotector.

**Table 1:** Physiological role of SIRT1

Physiological role of SIRT1	Ref.
1- SIRT1 preserves genomic stability during aging and regulates aging and cellular senescence.	(Chen et al., 2020).
2- SIRT1 suppresses the release of inflammatory mediators by downregulating NF-κB expression, thereby mitigating toxicant-induced inflammatory damage.	(Cui et al., 2022).
3- SIRT1 extends longevity by activating PGC-1α, thereby enhancing mitochondrial biogenesis.	(Yang et al., 2022).
4- SIRT1 promotes apoptotic cell death and improves protein stability by regulating insulin-like growth factor 1 (IGF-1) and PI3K signaling.	(Batiha et al., 2023; Xu et al., 2020; Yang et al., 2022).
5- SIRT1 increases antioxidant expression and reduces oxidative stress.	(Al-Kuraishy et al., 2023; Yang et al., 2022).
6- SIRT1 significantly reduced the inflammatory responses to IL-1β and TNF-α, which are augmented in aging.	(Orecchia et al., 2011).

## Aging Process: General Outlines

**Fig. 2:**
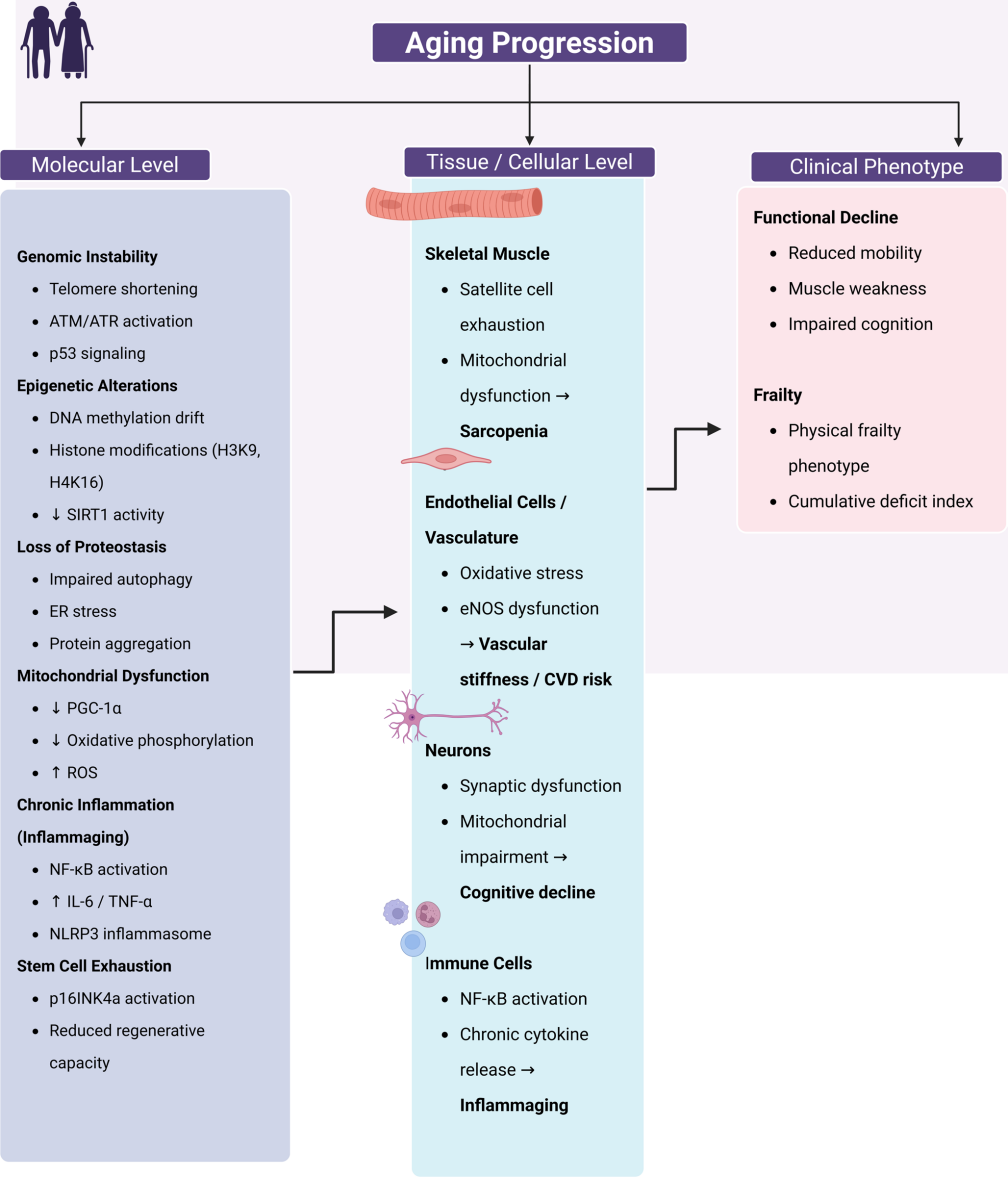
Molecular and cellular mechanisms underlying aging progression and frailty**.** Aging progression is driven by interconnected molecular alterations that culminate in tissue dysfunction and clinical frailty. At the molecular level, genomic instability (telomere shortening, ATM/ATR activation, p53 signaling), epigenetic alterations (DNA methylation drift, histone modifications including H3K9 and H4K16, and reduced SIRT1 activity), loss of proteostasis (impaired autophagy, endoplasmic reticulum stress, and protein aggregation), mitochondrial dysfunction (reduced PGC-1α signaling, impaired oxidative phosphorylation, and increased reactive oxygen species [ROS]), chronic inflammation (NF-κB activation, elevated IL-6 and TNF-α, and NLRP3 inflammasome activation), and stem cell exhaustion (p16INK4a activation and reduced regenerative capacity) collectively contribute to cellular decline. These molecular disturbances manifest at the tissue and cellular level in a cell-type–specific manner. In skeletal muscle, satellite cell exhaustion and mitochondrial dysfunction promote sarcopenia. In endothelial cells and the vasculature, oxidative stress and endothelial nitric oxide synthase (eNOS) dysfunction contribute to vascular stiffness and increased cardiovascular disease (CVD) risk. In neurons, synaptic dysfunction and mitochondrial impairment lead to cognitive decline. In immune cells, sustained NF-κB activation and chronic cytokine release drive inflammaging. The cumulative effects of these cellular dysfunctions result in clinical phenotypes characterized by functional decline (reduced mobility, muscle weakness, and impaired cognition) and frailty, including the physical frailty phenotype and increased cumulative deficit index. Together, these interconnected mechanisms illustrate the multilevel progression from molecular damage to organismal aging and frailty

The frequency of DNA damage increases with age, leading to genomic instability and an increased risk of mutations (Wang et al., 2021). Genome instability has long been implicated as the primary causal factor in aging. Somatic cells are continuously exposed to various sources of DNA damage, from ROS to UV radiation to environmental mutagens. However, DNA repair is erroneous, and such defects, as well as the occasional failure to correctly replicate the genome during cell division, are the basis for mutations and epimutations (Wang et al., 2021). There is now ample evidence that mutations accumulate in various organs and tissues of humans and animals. What is not known, however, is whether the frequency of these random changes is sufficient to cause the phenotypic effects generally associated with aging (Wang et al., 2021). Therefore, somatic tissues of aging organs have normal and mutant cells forming mosaicism (Vijg & Dong, 2020) (Figure 2).

**Fig. 3:**
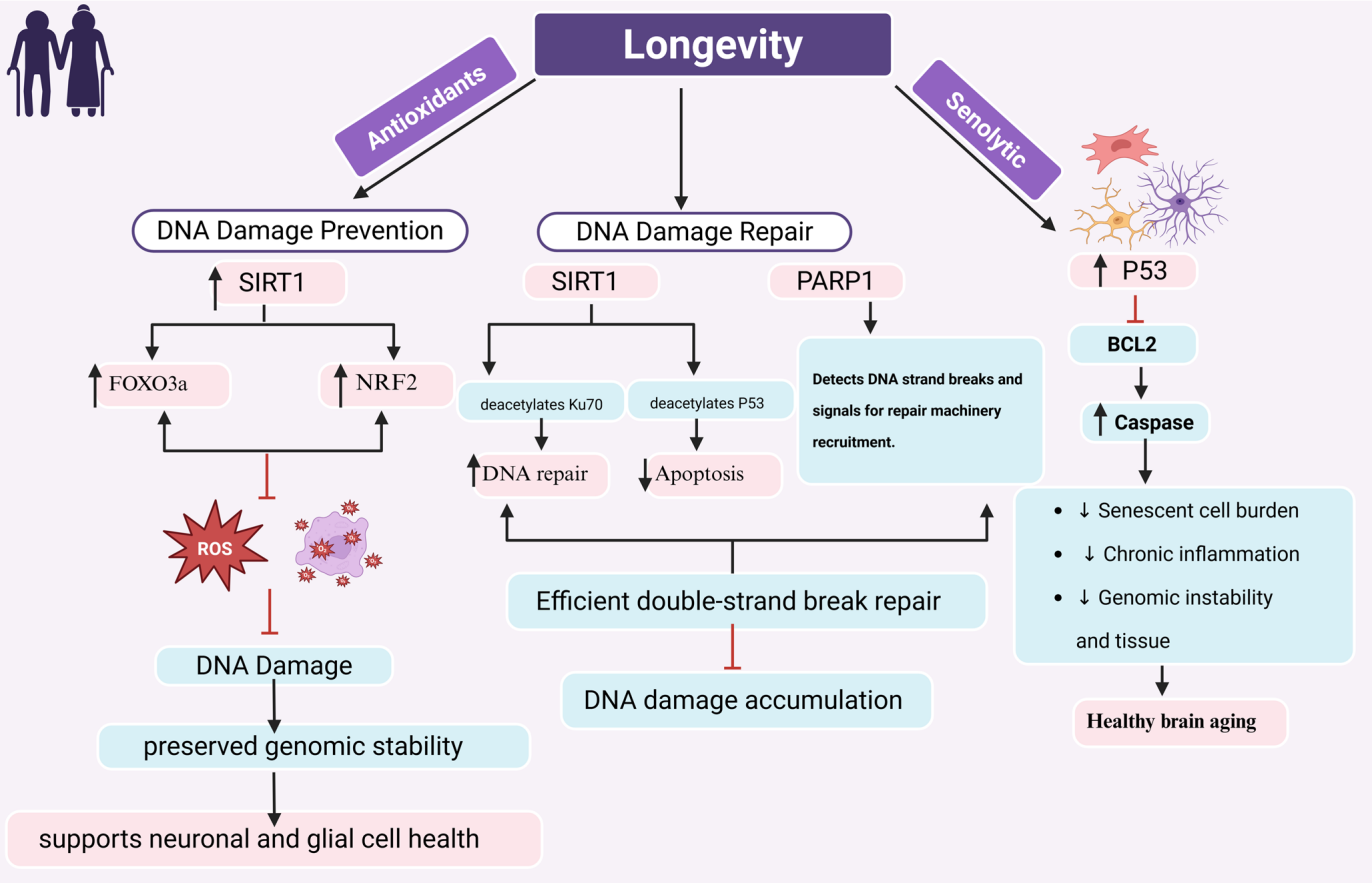
Mechanistic pathways linking DNA maintenance and senolytic processes to longevity. DNA damage prevention: SIRT1 activation enhances FOXO3a and NRF2 signaling, increasing antioxidant defenses, reducing ROS, and preserving genomic stability. DNA repair: SIRT1 deacetylates Ku70 and p53, while PARP1 detects DNA strand breaks, together promoting efficient double-strand break repair and limiting apoptosis. Elimination of dysfunctional cells: Senolytic pathways activate p53-mediated suppression of BCL2 and caspase-dependent apoptosis, reducing senescent cell burden, chronic inflammation, and genomic instability. Collectively, these mechanisms support neuronal and glial cell health, genomic integrity, and healthy brain aging

Of note, mechanisms such as DNA repair and the elimination of injured cells through apoptosis reduce DNA damage and the accumulation of mutant cells (Poetsch, 2020). However, these protective mechanisms are impaired during aging. Paradoxically, DNA repair is subjected to age-related changes and deterioration. For example, the efficiency of mismatch repair (MMR), base excision repair (BER), nucleotide excision repair (NER), and double-strand break (DSB) repair systems is impaired, leading to DNA mutations during aging (Gorbunova et al., 2007). Interestingly, endogenous antioxidants are the primary mechanism for reducing cell injury and macromolecular damage (Mumtaz et al., 2021). Depletion of endogenous antioxidant capacity and SIRT1 during aging promotes the development of oxidative stress, which triggers oxidative modifications of DNA and other macromolecules, resulting in various pathological processes such as neurodegeneration and carcinogenesis (Mumtaz et al., 2021). Therefore, the intensification of antioxidant defense mechanisms, the promotion of DNA repair, the elimination of dysfunctional aged cells (senescent cells), and the activation of the SIRT1 signaling pathway could be potential mechanisms for longevity (Wichansawakun & Buttar, 2019) (Figure 3).

In this context, agents that attenuate the development and progression of the aging process are called geroprotectors, which are senotherapeutics that affect the root causes of aging and related disorders by targeting senescent cells (Mbara et al., 2022). Senotherapeutics are commonly classified as senostatic (senomorphic), which inhibit the senescence-associated secretory phenotype (SASP), and senolytics, which trigger the death of senescent cells (Zhang et al., 2023). SASP is a group of molecules, including chemokines, pro-inflammatory cytokines, proteases, inhibitory molecules, bioactive lipids, extracellular vesicles, and other metabolites that induce chronic inflammation and cellular dysfunction (Kumari & Jat, 2021). In this context, SIRT1 may reduce aging and age-related disorders by inhibiting SASP expression (Zhang et al., 2023). SASP components affect surrounding cells and alter their microenvironment; SASP may be a key mechanism linking cellular senescence to individual aging and age-related diseases. Findings from a preclinical study showed that SIRT1 negatively regulated SASP factor expression at the transcriptional level during cellular senescence (Zhang et al., 2023), suggesting that SIRT1 repressed SASP factor expression by deacetylating histones at their promoter regions. Cellular senescence is activated by numerous stressors mediated by the aging process. Upon SASP release, senescent cells can modulate apoptosis and survival pathways (Schulz, 2023).

In normal tissues, proliferation of precursor cells is balanced by terminal differentiation and cell loss. Replicative senescence occurs after cells have undergone many replicative cycles or, more rapidly, in response to inappropriate proliferation signals. It is induced by TP53, specific cell cycle inhibitors, and RB1 (Schulz, 2023). Various forms of cell death, like apoptosis, can be elicited by stress, damage, inappropriate proliferation, or cytotoxic immune cells. Apoptosis can be initiated by two pathways that converge into a common execution cascade. The intrinsic pathway triggers the mitochondrial permeability transition, which ultimately leads to the activation of executor caspases. The extrinsic pathway is initiated by death receptors activated by cytokine ligands or cytotoxic immune cells; it can engage the intrinsic pathway for support. Diverse BH3 proteins stimulate, inhibit, or conduct apoptosis. Insufficient apoptosis in aged cells can result from inactivation of death receptor signaling, downregulation of pro-apoptotic factors, or upregulation of anti-apoptotic factors, driven by genetic or epigenetic alterations. Apoptotic ability is moreover compromised by TP53 inactivation or activation of PI3K and NFκB signaling (Schulz, 2023).

Moreover, senescent cells upregulate anti-apoptotic pathways, preventing them from dying despite the accumulation of damaged organelles and DNA. Therefore, cellular senescence may be a therapeutic target for aging and lifespan extension. However, senomorphics, such as NF-κB inhibitors, suppress senescent cells by inhibiting SASP. Nonetheless, senolytic agents promote apoptosis by targeting critical enzymes involved in pro-survival and pro-apoptotic signaling, such as p53 (Lagoumtzi & Chondrogianni, 2021). In addition, senotherapeutics may act as senolytic and senomorphic (Lagoumtzi & Chondrogianni, 2021). Senescent cells generally display heightened resistance to apoptotic signals. The development of first-generation senolytic agents was guided by hypothesis-driven, mechanism-based strategies that focused on disrupting anti-apoptotic pathways. Apoptosis is regulated by multiple factors, including B-cell lymphoma 2 (BCL-2) family proteins, heat shock proteins, and signaling pathways such as p53 and phosphatidylinositol 3-kinase/protein kinase B (PI3K/Akt). The senolytics ABT-737 and ABT-263 promote apoptosis in senescent cells by inhibiting BCL-2 family proteins (Lagoumtzi & Chondrogianni, 2021). Furthermore, natural flavonoids such as quercetin and fisetin function as senolytics by suppressing the PI3K/Akt survival pathway, whereas dasatinib exerts senolytic activity by targeting Src/tyrosine kinases (Wang et al., 2022). In particular, the combination of dasatinib and quercetin has been shown to eliminate p21-overexpressing cells in visceral adipose tissue (VAT) of obese individuals. In transplantation studies, mice receiving vehicle-treated human VAT developed glucose intolerance and insulin resistance. In contrast, those transplanted with VAT treated with dasatinib and quercetin remained insulin-sensitive and showed improvements in adverse metabolic outcomes (Wang et al., 2022). Moving to second-generation senolytics, these compounds are designed to target senescence-associated β-galactosidase (SA-β-gal), an enzyme that accumulates in senescent cells. For instance, the agent KSL0608-Se becomes phototoxic in the presence of SA-β-gal and selectively eradicates senescent cells in a light-dependent, dose-responsive manner (Shi et al., 2023).

## The Aging Process and Dysregulation of SIRT1 Signaling

It has been shown that during aging, SIRT1 levels are generally reduced, leading to increased oxidative stress; however, low levels of oxidative stress can even extend lifespan (Salminen et al., 2013). SIRT1 can stimulate antioxidant gene expression via the FoxO pathways and inhibit NF-κB signaling, a significant inducer of inflammatory responses. Remarkably, an increased level of ROS can both directly and indirectly control the activity of the SIRT1 enzyme. For instance, ROS can inhibit SIRT1 activity by evoking oxidative modifications on its cysteine residues. Decreased SIRT1 activity enhances NF-κB signaling, which supports inflammatory responses. This crosstalk between SIRT1 and ROS signaling provokes, in a context-dependent manner, a decline in autophagy and a low-grade inflammatory phenotype, both common hallmarks of ageing (Salminen et al., 2013).

Preclinical studies have shown a progressive decline in SIRT1 expression in the brains of aged mice (Diaz-Perdigon et al., 2020). Since epigenetic changes are key contributors to aging, SIRT1, like SIRT2, may counteract age-related cognitive decline and neuropathology, as demonstrated in SAMP8 mice (Diaz-Perdigon et al., 2020). The age-related reduction of SIRT1 is even more pronounced in models of accelerated aging (Sasaki et al., 2006). In prematurely senescent cells, SIRT1 decreases rapidly and shows an inverse correlation with SA-β-gal activity. Both in mouse and human fibroblasts, SIRT1 expression is positively correlated with proliferating cell nuclear antigen (PCNA), a DNA replication factor expressed during S-phase (Sasaki et al., 2006). In animals, age-related declines in SIRT1 are most evident in tissues with reduced mitotic activity, such as the thymus and testes, but not in tissues like the brain, where mitotic activity remains relatively stable (Sasaki et al., 2006). These reductions in SIRT1 closely parallel decreases in PCNA levels. Interestingly, while SIRT1 loss is accelerated in mice with premature aging, it is absent in long-lived growth hormone receptor knockout mice (Sasaki et al., 2006). Therefore, as mitotic activity ceases in mouse and human cells in the typical environment of the animal or the culture dish, SIRT1 levels decline concomitantly. Aging-induced SIRT1 loss is tissue-specific; it is gradually reduced in the brain (Gong et al., 2014). Therefore, different life stages, including young age before maturation, adulthood, middle age, and old age, are associated with SIRT1 expression, which declines with age at the transcriptional and translational levels in the brain, liver, skeletal muscle, and white adipose tissue in SAM-P8 and SAM-R1. SIRT1 expression is lower in SAM-P8 than in SAM-R1, particularly at old age (Gong et al., 2014). A prospective cohort study in men aged 70 years and older found that serum-induced SIRT1 expression was not associated with age or mortality. Instead, it correlated with markers of body composition and nutritional status, including height, weight, body fat percentage, lean mass, albumin, and cholesterol, but showed no association with disease (Razi et al., 2017). Similarly, SIRT1 SNPs rs2273773, rs3740051, and rs3758391 were not associated with age, frailty, or mortality, but were associated with weight, height, body fat, lean mass, and albumin levels. No relationship was observed between these SNPs and serum-induced SIRT1 expression (Razi et al., 2017). Therefore, SIRT1 and serum-induced SIRT1 protein levels are mainly associated with nutritional status rather than aging. In addition, SIRT1 activity reflects metabolic status rather than the aging process (Lee & Yang, 2017).

Correlation analysis of the retrospective study showed that SIRT1 activity is significantly correlated with serum triglyceride levels in men and with waist circumference, systolic blood pressure, diastolic blood pressure, and serum triglyceride levels in women. Positive correlation was observed between SIRT1 activity and BMR in women but not in men (Lee & Yang, 2017), suggesting that serum SIRT1 activity may be a biomarker of aging. In addition, a positive correlation between SIRT1 activity and BMR in women indicates that serum SIRT1 activity may reflect energy expenditure well in humans. These findings highlight the sex-related effect of SIRT1. Conversely, a cohort study found that SIRT1 levels are elevated with age, particularly in subjects with the SIRT1 rs7895833 polymorphism, suggesting a compensatory role for SIRT1 in mitigating age-related oxidative stress (Woźny et al., 2017). A clinical finding revealed that SIRT1 expression is reduced in elderly patients with chronic heart failure due to upregulation of pro-apoptotic p53 (Lu et al., 2014). Thus, in advanced heart failure, reduced SIRT1 expression, similar to age-related changes, may contribute significantly to decreased antioxidant activity and increased expression of pro-apoptotic molecules via the p53, FoxO1, and oxidative stress pathways (Lu et al., 2014). These observations underscore the decline in SIRT1 signaling as a hallmark of the aging process.

## The Brain Aging Process: An Overview

Brain function progressively deteriorates with aging due to functional and structural neuronal changes, including morphological changes in dendritic spines and dysfunction of synaptic plasticity/neurotransmission (Melo Dos Santos et al., 2024). Brain aging manifests in cognitive impairment, memory disorders, attention deficit, and visuospatial disorientation (Habes et al., 2021). Neuronal senescence is the primary pathway in the progression of brain aging (Sahu et al., 2022). Remarkably, senescence neurons have a low capacity to undergo apoptosis, exhibit impaired autophagy, yet show augmented SAβ-gal activity (Kavanagh et al., 2021; Sah et al., 2021). Cellular senescence is now understood as a multifaceted stress response that alters cell fate. Rather than responding to cellular or DNA damage with proliferation or apoptosis, senescent cells persist in a permanent state of cell cycle arrest. These cells also contribute to chronic tissue decline by releasing harmful factors that negatively influence neighboring cells (Sah et al., 2021). In neuroscience, the concept of senescence has been extended to the brain, even to post-mitotic cells, a notion that initially posed challenges for senescence researchers. Nevertheless, growing investigations into the role of senescence in brain aging, neurodegenerative diseases, and injury are driving progress in this field (Sah et al., 2021).

It has been shown that biomarkers of the senescent cells are increased during brain aging (Kavanagh et al., 2021). For example, circulating plasminogen activator inhibitor-1 (PAI-1), the only inflammation-related biomarker found to increase with age, is considered an indicator of senescent cell burden (Kavanagh et al., 2021). Findings from a preclinical study revealed that PAI-1, a serine protease inhibitor, is upregulated and correlates with increased expression of cell cycle repressors p53 and p21 in the hippocampus/cortex of senescence-accelerated mouse prone 8 (SAMP8) mice and AD patients. Double immunostaining results show that astrocytes in the brains of AD patients and SAMP8 mice express higher levels of senescent markers and PAI-1 than those in the corresponding controls (Kavanagh et al., 2021), suggesting that increased PAI-1 may contribute to brain cell senescence in AD. Senescent astrocytes can induce neuron apoptosis through secreting pathologically active molecules, including PAI-1. Moreover, extensive evaluation of adipose SAβ-gal staining in a relevant animal model of human aging supports the validity of PAI-1as a biomarker of age-associated processes (Kavanagh et al., 2021).

**Fig. 4:**
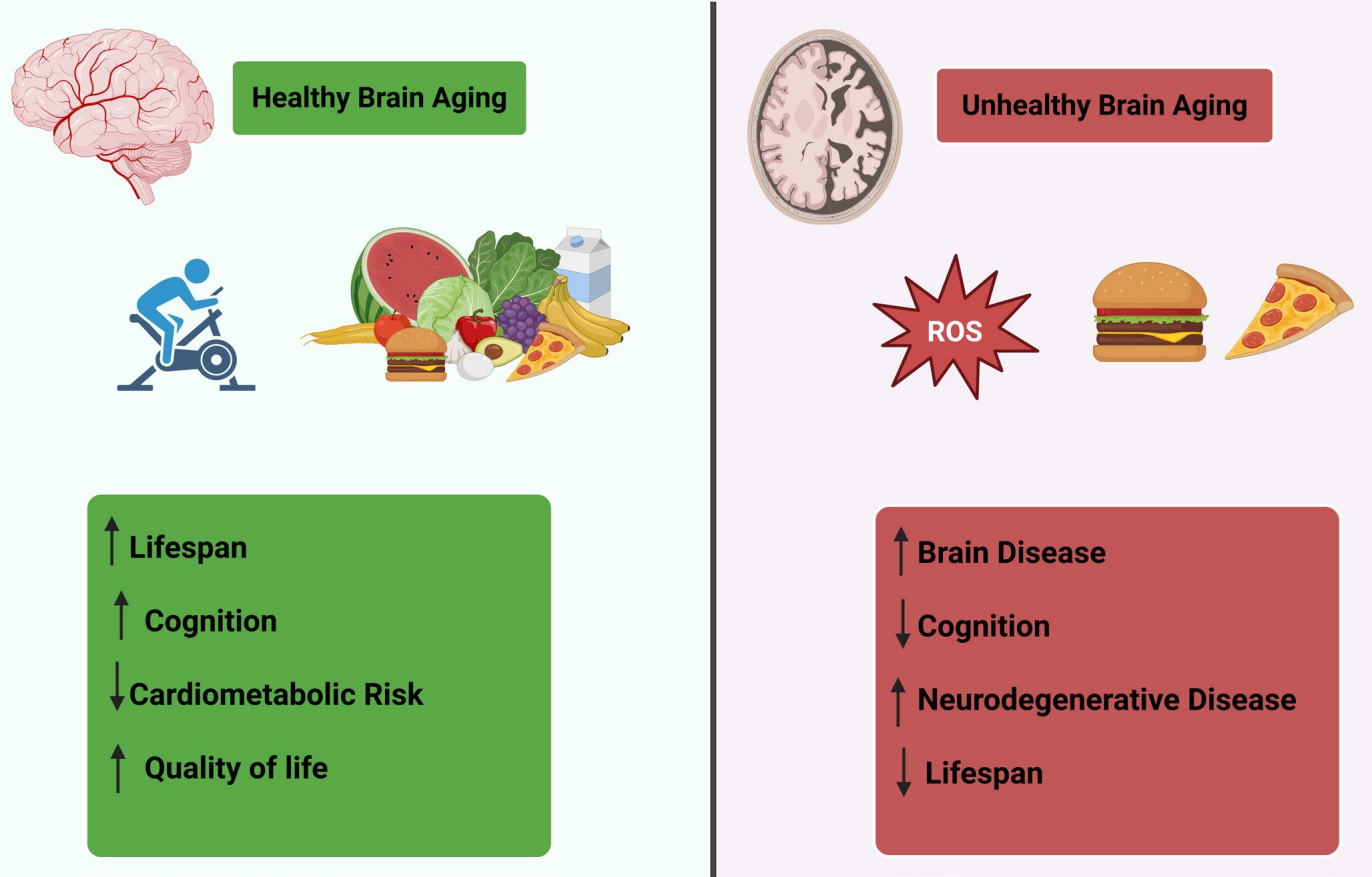
Healthy and unhealthy brain aging. Healthy brain aging, supported by regular physical activity, balanced nutrition, and the control of oxidative stress, is associated with improved cognition, extended lifespan, and reduced cardiometabolic risk. In contrast, unhealthy brain aging, driven by poor dietary habits and elevated reactive oxygen species (ROS), promotes oxidative damage, neurodegenerative pathology, cognitive decline, and shortened lifespan. Lifestyle and nutritional factors are central to maintaining neuronal integrity and modulating oxidative stress during aging

Remarkably, neuronal senescent cells resist apoptosis and depend mainly on autophagy for their metabolic functions. The heterogeneity of senescent cells indicates that they rely on different proteins or pathways for survival. Therefore, a better understanding of the underlying mechanisms for apoptotic resistance of senescent cells will provide new molecular targets for the development of cell-specific or broad-spectrum therapeutics to clear senescent cells (Hu et al., 2022). As the brain ages, its functional capacity progressively declines, and cellular senescence contributes to aging and is a hallmark of many age-related conditions. The transcriptional repressor known as RE1-silencing transcription factor (REST) has been linked to aging and plays a crucial role in protecting neurons from developing a senescent phenotype (Rocchi et al., 2021). Loss of REST function disrupts autophagy and proteostasis, elevates oxidative stress, and increases neuronal death. Thus, restoring autophagy function can counteract key features of senescence (Rocchi et al., 2021). Therefore, REST contributes to healthy brain aging by maintaining autophagic activity and regulating the senescence program in neurons, processes relevant to neurodegenerative disorders associated with aging. Healthy brain aging involves a combination of physical activity, good nutrition, staying mentally and socially engaged, managing health conditions (e.g., blood pressure, diabetes), getting enough quality sleep, and avoiding processed foods to slow cognitive decline and maintain function, distinguishing regular, subtle changes from dementia (Gronek & Tang, 2023). Conversely, unhealthy brain aging involves accelerated cognitive decline beyond regular changes, characterized by faster processing, significant memory loss, difficulties with problem-solving, and behavioral issues, often linked to lifestyle factors like poor diet, lack of sleep, stress, inactivity, and vascular problems that increase risk for neurodegenerative diseases. Unhealthy aging is characterized by more severe declines due to factors such as white matter lesions, protein buildup (amyloid), and reduced neurotransmitter levels, accelerating disease risk (Peter et al., 2025) (Figure 4**).**

Furthermore, glial cells tend to undergo senescence, leading to increased release of pro-inflammatory cytokines and the development of neuroinflammation. Senescent glial cells exacerbate neuroinflammation, disrupt synaptic function, and promote neuronal death (Alshaebi et al., 2025). Indeed, age-related memory and cognitive impairment are associated with brain changes, particularly in the hippocampus and prefrontal cortex (Jobson et al., 2021). These neuropathological alterations increase the risk of developing neurodegenerative disorders (Jobson et al., 2021; Zhang et al., 2019). It has been shown that gene expression of brain neurotransmitters and neuronal proteins required for synaptic plasticity and cognitive function is reduced after 40 years, leading to cognitive impairments (Aquilani et al., 2023). The hallmarks for neuronal aging are impairment of the clearance system, accumulation of oxidized proteins, reduction of neuronal energy metabolism, and abnormal adaptive stress response (Błaszczyk, 2020). Also, alterations in synaptic neurotransmission and the development of aberrant synaptic function are commonly recognized in the aged brain (Nagy et al., 2021). Moreover, the neuroprotective polyamine spermidine, which improves synaptic plasticity and has anti-aging effects, is highly reduced in the aging brain (Chen et al., 2020).

## The Neuroprotective Role of SIRT1 Signaling in Brain Aging

SIRT1, the well-studied mammalian sirtuin, functions across various tissues and organs, but its influence on aging and lifespan is particularly linked to its role in CNS neurons (Batiha et al., 2023; Yang et al., 2022). SIRT1 regulates energy metabolism, circadian rhythm, supports dendritic and axonal growth, and enhances neuronal resilience to stress. SIRT1 is also essential for maintaining plasticity, cognitive performance, and protection against age-related neurodegeneration and cognitive decline (Batiha et al., 2023; Yang et al., 2022). Furthermore, SIRT1 plays a critical role in regulating brain function. It has a neuroprotective effect against brain aging and neurodegenerative diseases by activating neurogenesis and modulating neuritogenesis, the outgrowth of axons and dendrites (Herskovits & Guarente, 2014). Herskovits and Guarente found that SIRT1 is necessary for brain development and control of brain senescence (Herskovits & Guarente, 2014). SIRT1 promotes axonal growth, dendritic branching, and neurite outgrowth, and enhances memory function by activating synaptic plasticity (Herskovits & Guarente, 2014).

In the human brain, SIRT1 is abundantly expressed and contributes to enhanced neurogenesis and neuronal myelination (Mormone et al., 2023). It plays a pivotal role in axonal development and dendritic function, supporting synaptic plasticity and cognitive performance while protecting against age-related neurodegeneration and cognitive decline (Godoy et al., 2014; Ng et al., 2015). Notably, SIRT1 mRNA is abundantly expressed in brain regions that regulate energy balance and metabolic activity, such as the hypothalamus, the nucleus tractus solitarius, and pro-opiomelanocortin (POMC) neurons (Ramadori et al., 2008). However, SIRT1 protein levels specifically rise in the hypothalamus during fasting, a response that is disrupted in leptin-deficient obese mice (Ramadori et al., 2008). Beyond the metabolically relevant regions, SIRT1 mRNA is also abundantly expressed in the piriform cortex, hippocampus, medial habenular nucleus, and cerebellum (Ramadori et al., 2008).

Remarkably, activation of the hippocampal SIRT1 signaling pathway improves cognitive function in transgenic mice by enhancing proteostasis and activating neurotrophic factors (Corpas et al., 2017). Likewise, the SIRT1 signaling pathway is an essential protective pathway against hippocampal atrophy and the cognitive impairment it induces during aging (Sun et al., 2022). Therefore, they may be potential therapeutic targets for preventing and intervening in aging-related neurodegenerative diseases. SIRT1 acts as a central NAD⁺-dependent metabolic sensor that integrates multiple signaling pathways involved in brain aging. Age-related decline in NAD⁺ availability reduces SIRT1 activity, contributing to mitochondrial dysfunction, impaired antioxidant defense, and chronic neuroinflammation. In neurons, SIRT1 regulates mitochondrial biogenesis, synaptic plasticity, and survival signaling through pathways such as AMPK/PGC-1α and Akt/mTOR.

In contrast, in glial cells, particularly microglia, SIRT1 primarily modulates neuroinflammatory responses by suppressing NF-κB-mediated transcription of pro-inflammatory cytokines. Therefore, the NAD⁺–SIRT1 axis represents a unifying mechanistic framework linking metabolic regulation, inflammatory signaling, and neuronal resilience during brain aging. SIRT1 modulates inflammatory responses by deacetylating histones and critical transcription factors, such as activator protein 1 and NF-κB, thereby blocking the transcription of specific genes that promote inflammation. SIRT1 also supports vascular endothelial cells by regulating antioxidant genes via a FoxO3a/PGC-1α complex (Cao et al., 2018). Evidence supports SIRT1’s involvement in aging and its interactions with several signaling pathways, including NF-κB, AMPK, mTOR, p53, PGC1α, and FoxOs (Chen et al., 2020). Notably, SIRT1 regulates several processes, including the inflammatory response, apoptosis, oxidative stress, energy metabolism, and autophagy, through deacetylase activity and NF-κB signaling. As the center of multiple intracellular signaling pathways, NF-κB activity is regulated by multiple factors. SIRT1 can both directly deacetylate NF-κB and, indirectly through other molecules, inhibit its activity (Liu et al., 2024). Findings from preclinical study illustrated that ginsenoside Rg3 is effective in reducing neuroinflammation and damage in hippocampal neurons subsequent traumatic brain injury by modulating the SIRT1/NF-kB pathway signifying its possible as a therapeutic agent for traumatic brain injury (Liu et al., 2024). The SIRT1/NF-kB pathway is crucial in the regulation of brain injury and it has been shown that certain medications can mitigate brain injury by activating SIRT1 and deactivating NF-kB (Song & Zhou, 2022). Moreover, SIRT1 regulates the expression of several antioxidant genes such as superoxide dismutase, catalase, peroxiredoxins 3 and 5 (Prx3, Prx5), thioredoxin 2 (Trx2), thioredoxin reductase 2 (TR2), and uncoupling protein 2 (UCP-2) by upregulating the FoxO3a/PGC-1α complex (Olmos et al., 2013). Gupta et al. illustrated that FOXO transcription factors, regulated by SIRT1 deacetylation, enhance antioxidant defense mechanisms, thereby counteracting oxidative stress and metabolic dysregulation in brain aging. Moreover, key redox-sensitive regulators such as Nrf2 and PGC-1α interact with this pathway, arranging mitochondrial biogenesis and adaptive stress responses (Gupta et al., 2025). In addition, the longevity-related AMPK/SIRT1/PGC-1α signaling pathway and brain IGF1/PI3K/Akt survival pathway are significantly reduced in brain aging, and increased after exercise training in animal models signifying that exercise not only reduced aging-induced brain apoptosis and inflammatory signaling activity, but also enhanced the survival pathways in the hippocampus, which provides one of the new beneficial effects for exercise training in aging brain (Lin et al., 2020). Primary neurons derived from transgenic mice overexpressing SIRT1 exhibit greater neurite outgrowth and survival, primarily due to the negative regulation of mammalian/mechanistic target of rapamycin (mTOR) signaling. SIRT1 localizes at the axonal growth cone, where its activation promotes axon formation and elongation, likely through AKT deacetylation (Li et al., 2013).

Furthermore, SIRT1 has neuroprotective effects against the development and progression of neurodegenerative diseases such as Parkinson’s disease (PD) and Alzheimer’s disease (AD) (Batiha et al., 2023). AD is the most common neurodegenerative disease due to the progressive accumulation of Aβ and hyperphosphorylated tau protein. AD is an age-related disease characterized by cognitive impairment and memory loss (Al-Kuraishy et al. 2025a, b). Senolytic therapy in AD mouse models has been shown to selectively eliminate senescent cells from the plaque microenvironment, leading to reduced neuroinflammation, decreased Aβ burden, and improvements in cognitive performance (Zhang et al., 2019). Preclinical evidence further indicates that senescent cells progressively accumulate with age across multiple organ systems (Zhang et al., 2019). In the brain, tau protein aggregation is strongly linked to cognitive deterioration in AD and other tauopathies, and it appears to drive cellular senescence (Al-Kuraishy et al. 2025a, b). Pharmacological removal of senescent cells in tauopathy mouse models alleviates disease pathology. Compared with vehicle-treated controls, mice given intermittent senolytic treatment showed reduced tau accumulation and neuroinflammation, preserved neuronal and synaptic density, normalized cerebral blood flow, and diminished ventricular enlargement. Importantly, intermittent administration of dasatinib plus quercetin has demonstrated a favorable safety profile in clinical studies targeting other senescence-related conditions (Gonzales et al., 2022). An initial open-label clinical trial pilot illustrated that an intermittent senolytic combination therapy of dasatinib plus quercetin in five older adults with early-stage AD. The primary objective is to evaluate the CNS penetration of dasatinib and quercetin by analyzing CSF collected at baseline and after 12 weeks of treatment; further, through a series of secondary outcome measures to assess target engagement of the senolytic compounds and AD-relevant cognitive and functional (Gonzales et al., 2022). Moreover, reduced levels of SIRT1 in the brains of patients with AD may be related to the decline in SOD-1 and neuropathological changes of this disorder. The number of both SIRT1-positive and SOD-1-positive neurons and the integrated optical density of immunohistochemical staining for these proteins in the temporal and frontal cortices and the hippocampus of patients with AD were significantly decreased compared with those in corresponding controls. In the cerebellum, very weak SIRT1 expression and strong SOD-1 expression were observed in granule cells, with no significant difference between the AD and control groups. Interestingly, the levels of SIRT1 and SOD-1 are dysregulated in all regions of the AD brains investigated, except the cerebellum (Cao et al., 2018).

Furthermore, PD is a progressive neurodegenerative disease characterized by the accumulation of mutant alpha-synuclein (α-Syn) and the degeneration of dopaminergic neurons in the substantia nigra pars compacta (SNpc) (Miller et al., 2023). It has been emphasized that senolytic and senomorphic agents improve PD neuropathology in *D. melanogaster* (Miller et al., 2023). Findings from a preclinical study demonstrated that α-Syn pathology could lead to astrocyte and/or microglia senescence in PD brains, which may contribute to neuropathology in this model (Verma et al., 2021). Therefore, targeting senescent cells using senolytics could constitute a viable therapeutic option for treating PD.

Significantly, SIRT1 influences brain-derived neurotrophic factor (BDNF) expression by deacetylating methyl-CpG binding protein 2 (MeCP2), a transcription factor implicated in Rett syndrome. This deacetylation promotes MeCP2 release from the BDNF promoter, thereby elevating BDNF transcription and release (Wang et al., 2023). Elevated SIRT1 expression, particularly in hippocampal neurons, may thus support synaptic plasticity by regulating dendritic spine remodeling and connectivity. Interestingly, BDNF attenuates the brain aging process and the development of neurodegenerative diseases. Findings from a preclinical study showed that decreased BDNF-TrkB signaling during aging favors microglial activation.

In contrast, upregulation of BDNF signaling inhibits microglial activation via the TrkB-Erk-CREB pathway in mice (Wu et al., 2020). As well, BDNF sequentially kinetic activated (SKA) counteracts mechanisms underlying CNS degeneration and aging by increasing endogenous protective mechanisms. Both in vitro and in vivo experiments demonstrated that BDNF SKA induces endogenous BDNF production via its receptor TrkB and influences ApoE4 expression. Moreover, BDNF SKA exerted effects on β-Amyloid and SIRT1 proteins, confirming the hypothesis of a fine endogenous regulatory role in maintaining the health of both neurons and astrocytes (Molinari et al., 2020). For this reason, a change in BDNF turnover is considered a positive factor in the fight against brain aging. Significantly, senolytic agents that eradicate senescent neurons can improve cognitive function. Senolytic treatment of AD mice selectively removed senescent cells from the plaque environment, reduced neuroinflammation, lowered Aβ load, and ameliorated cognitive deficits [49], suggesting a potential role for senolytic agents in aging and aging-related neurodegeneration. These findings indicated that impairment of neuroprotective SIRT1 signaling contributes to the brain aging process (Table 2).

**Table 2:** The neuroprotective role of SIRT1 signaling against brain aging

Studies	Findings	Ref.
Preclinical	SIRT1 preserves synaptic plasticity, cognitive performance, and protection against age-related neurodegeneration and cognitive decline.	(Batiha et al., 2023; Yang et al., 2022).
Preclinical	SIRT1 activates neurogenesis and modulates neuritogenesis.	(Herskovits & Guarente, 2014).
Preclinical	SIRT1 activates neurite outgrowth and survival by negatively regulating mTOR signaling.	(Li et al., 2013).
Preclinical	Hippocampal SIRT1 improves cognitive function in transgenic mice by enhancing proteostatic mechanisms and neurotrophic factor signaling.	(Corpas et al., 2017).
Preclinical	SIRT1 signaling pathway prevents hippocampal atrophy and cognitive impairment during aging.	(Sun et al., 2022).
clinical	SIRT1 and SOD-1 are dysregulated in all regions of the AD brains investigated except for the cerebellum.	(Cao et al., 2018)
Preclinical	SIRT1 influences BDNF expression by deacetylating methyl-CpG binding protein 2 (MeCP2), a transcription factor implicated in Rett syndrome.	(Wang et al., 2023).

## Dysregulation of SIRT1 Signaling in Brain Aging

Furthermore, SIRT1 is highly dysregulated in the aging brain and related processes. SIRT1 has an anti-aging effect by deacetylating different proteins involved in cellular pathways such as apoptosis and stress response (Chen et al., 2020). In mice, SIRT1 expression declines with age, whereas enhanced SIRT1 activity has been shown to prolong lifespan in both yeast and mice. Recently, considerable attention has been given to how external factors, such as polyphenol dietary compounds with epigenetic effects, can regulate molecular processes linked to aging. These natural agents influence cellular longevity by modifying histones post-translationally and by promoting autophagy, which reduces acetyl coenzyme A (AcCoA) availability. A pilot study showed that plasma SIRT1 levels were lower in healthy elderly volunteers than in healthy controls (Libri et al., 2012). SRT2104 has been developed as a selective small molecule activator of SIRT1, which regulates energy homeostasis and modulates various metabolic pathways, including glucose metabolism, oxidative stress, and lipid metabolism (Libri et al., 2012).

A pilot randomized, placebo-controlled, double blind phase I trial showed that SRT2104 was generally safe and well-tolerated. Pharmacokinetic exposure increased less than dose-proportionally with increased mitochondrial oxidative phosphorylation (Libri et al., 2012). In addition, plasma SIRT1 levels are reduced in elderly patients with ischemic stroke, a common CNS disorder in aging (Esmayel et al., 2021). Plasma SIRT1 levels are lower in patients with acute cerebrovascular stroke than in controls. Also, SIRT1 may be a possible biomarker for predicting the risk of acute cerebrovascular stroke. Notably, in healthy individuals, plasma SIRT1 concentrations correlate with age, body mass index, indicators of carbohydrate metabolism, and markers of antioxidant status. A comparison between individuals with accelerated aging and those aging normally revealed significant differences in SIRT1 plasma levels (Kolesnikova et al., 2023). Nonetheless, this study did not establish a link between SIRT1 gene polymorphisms and variations in plasma SIRT1 levels, aging pace, or metabolic traits. Substantial research has shown that oxidative stress and inflammation are major contributors to age-related degeneration in peripheral tissues. Yet, there is relatively little evidence clarifying their role in the normal aging process of the brain. Elevated oxidative damage, however, has been associated with critical cellular alterations, including diminished NAD availability (Guest et al., 2014). In addition, progressive aging is associated with increased oxidative damage, inflammation, and reduced NAD in the brain (Guest et al., 2014).

Furthermore, CSF SIRT1 levels are lower in elderly patients than in young subjects (Guest et al., 2014). Additionally, it has been shown that microglial SIRT1 deficiency is associated with memory impairment and accelerated aging in mice, due to upregulation of the inflammatory response (Guest et al., 2014). Chronic inflammation and aging microglia contribute to neurodegenerative cognitive deficits. Consistently, a decline in SIRT1 accompanies microglial aging, and loss of SIRT1 in microglia contributes to aging-related processes and tau-associated memory impairments through increased IL-1β expression in mice (Cho et al., 2015). This selective rise in IL-1β transcription may result from hypomethylation at specific regions of its proximal promoter caused by SIRT1 deficiency. In humans, IL-1β hypomethylation is closely associated with advancing chronological age and increased IL-1β gene expression (Guest et al., 2014). Thus, aging-induced SIRT1 deficiency in the brain contributes to the development and progression of cognitive decline and memory deficits [Table 3].

Therefore, the aging process distorts the brain SIRT1 signaling pathway, leading to cognitive impairment and neurodegeneration. Thus, SIRT1 activators may restore and reverse brain aging.

**Table 3:** Dysregulation of the brain SIRT1 signaling pathway in brain aging

Study type	Findings	Ref.
Preclinical	In mice, SIRT1 expression declines with age, whereas enhanced SIRT1 activity has been shown to prolong lifespan via interactions with several signaling pathways, including NF-κB, AMPK, mTOR, p53, PGC1α, and FoxOs.	(Chen et al., 2020).
Clinical	Plasma SIRT1 levels are lower in healthy elderly volunteers than in healthy controls.SIRT1 activator SRT2104 regulates brain energy homeostasis and modulates various metabolic pathways, including glucose metabolism, oxidative stress, and lipid metabolism.	(Libri et al., 2012).
Clinical	Plasma SIRT1 level is reduced in elderly patients with ischemic stroke, and may be a possible biomarker for predicting the risk of acute cerebrovascular stroke.	(Esmayel et al., 2021).
Clinical	CSF SIRT1 levels are lower in elderly patients than in young subjects.	(Guest et al., 2014).
Preclinical	A decline in SIRT1 is associated with microglial aging, and loss of SIRT1 in microglia contributes to aging-related processes and tau-associated memory impairments through increased IL-1β expression in mice.	(Cho et al., 2015)

## The Therapeutic Role of SIRT1 Activators in Brain Aging

### Pharmacological SIRT1 Activators

SIRT1 activators, synthetic or natural compounds, act as allosteric stimulators of SIRT1 activity. Brain SIRT1 overexpressing mice have an extended lifespan by delaying the aging process (Satoh et al., 2013). These phenotypes are mediated by enhanced neural activity in the dorsomedial and lateral hypothalamic nuclei, driven by increased expression of orexin type 2 receptors. Findings from a preclinical study revealed that SIRT1 upregulates transcription of the orexin type 2 receptor and colocalizes with SIRT1 in the dorsomedial and lateral hypothalamic nuclei (Satoh et al., 2013). Hence, SIRT1 in the dorsomedial and lateral hypothalamic nuclei regulates aging and longevity in mammals. In addition, many studies have shown that SIRT1 activators mimic endogenous SIRT1 activity (Liu et al., 2023). SIRT1 activators recapitulate many of the molecular events downstream of calorie restriction in vivo, such as enhancing mitochondrial biogenesis, improving metabolic signaling pathways, and blunting pro-inflammatory pathways in mice fed a high-fat, high-calorie diet (Smith et al., 2009). Moreover, SIRT1 activator SRT1720 was shown to suppress the production of inflammatory cytokines and cellular senescence in humans (Sung et al., 2021). Moreover, SRT1720 was shown to suppress the production of inflammatory cytokines and cellular senescence in human dermal fibroblasts by deacetylating acetyl-NF-κB and increasing the expression of both autophagy-related proteins and SIRT1 (Sung et al., 2021). In aged mice treated with SRT1720, higher levels of SIRT1 and autophagy-associated proteins were observed compared to untreated counterparts, along with reduced acetyl-NF-κB, inflammatory cytokines, and senescence markers (Sung et al., 2021). Therefore, SIRT1 activators may be a promising therapeutic strategy against brain aging.

#### Resveratrol

Resveratrol is a phenolic compound derived from the flowering plant Veratrum grandiflorum and is used as a remedy for various diseases (de la Lastra & Villegas, 2005). Resveratrol is found in many plants, such as grapes, peanuts, and berries. Resveratrol exerts anti-inflammatory and antioxidant effects through mechanisms including SIRT1 activation, inhibition of inducible nitric oxide synthase, cyclooxygenase-2 (COX-2), NF-κB, and activated protein 1. Resveratrol can extend lifespan in various organisms, particularly in mice on a high-calorie diet (de la Lastra & Villegas, 2005). Resveratrol has potent anti-aging and associated complications (Kasiotis et al., 2013). It has been suggested that Resveratrol could be effective in preventing aging and linked neurodegenerative diseases. The anti-aging mechanisms of resveratrol primarily involve reducing oxidative stress, modulating inflammatory responses, improving mitochondrial function, and regulating apoptosis. Resveratrol could be an effective and safe compound for preventing and treating aging and age-related diseases (Zhou et al., 2021).

Resveratrol is essential in regulating brain functions, such as cognitive and memory functions, by regulating synaptic plasticity. It reduces the risk of brain aging by attenuating oxidative stress and inflammation and activating the neuronal SIRT1 signaling pathway (Pyo et al., 2020). Resveratrol modulates signaling pathways involved in brain aging, including the SIRT1 pathway. The most essential processes in aging-associated neurodegeneration conditions are the mTOR, SIRT1, and insulin/insulin growth factor 1 signaling pathways. These longevity pathways involve multiple mechanisms, including metabolism, cognition, the stress response, and brain plasticity (Mazucanti et al., 2015). In yeast, low doses of resveratrol increase lifespan by 70%, whereas higher doses have minimal effects (Howitz et al., 2003). In addition, resveratrol interacts with many other proteins, including AMPK, complex III of the mitochondrial electron transport chain, PARP1, and phosphodiesterase (Bonkowski & Sinclair, 2016). A systematic review and meta-analysis found that resveratrol has a life-extending effect (Hector et al., 2012). In addition, resveratrol reduces the pathogenesis of age-related neurodegenerative diseases, including AD and PD (Pyo et al., 2020). Resveratrol also inhibits inflammaging, an exaggerated immune response to brain inflammatory reactions during aging (Sarubbo et al., 2018). Inflammaging is suggested as an essential mechanism in the development of brain aging (Gordleeva et al., 2020). Moreover, resveratrol attenuates cellular senescence in human cells by inhibiting the mTOR signaling pathway (Demidenko & Blagosklonny, 2009). Flores et al. (Flores et al., 2016) suggest that resveratrol promotes neuronal connectivity by inhibiting neuronal senescence during aging. The precise mechanism of resveratrol appears to be senomorphic, activating the SIRT1 signaling pathway in brain neurons (Ksila et al., 2023). However, at higher concentrations, resveratrol induces apoptosis of senescent neurons (Zhang et al., 2023). The antiaging effects of polyphenols could be due to several mechanisms, including the prevention of oxidative stress, activation of SIRT1, and modulation of inflammaging via regulation of signaling pathways, such as NF-κB (Zhang et al., 2023). Therefore, resveratrol has dual senolytic and senomorphic effects on senescent neurons in the aging brain by activating the SIRT1 signaling pathway.

Conversely, most polyphenols, including resveratrol, appear to have very low bioavailability from oral administration, yet many studies show beneficial health effects through this route. The main obstacle to this low bioavailability appears to be its rapid metabolism, although a still-controversial question is whether its metabolites retain therapeutic properties. In addition, it has been reported that some polyphenols are retained in neural tissue, reaching concentrations higher than those in plasma (Almeida et al., 2016). Much of the relevance of polyphenols in protecting the brain against aging stems from their ability to cross the BBB and their lipophilic nature (Liu et al., 2011). Polyphenols affect a wide range of mechanisms in the brain that help to protect against aging, improving cognition, exploratory behavior, spatial learning, and memory (Almeida et al., 2016; Liu et al., 2011). Therefore, resveratrol contributes to preserving mental health, as it reduces the risk of dementia and prevents the onset of neurodegenerative diseases during aging.

Nevertheless, open questions hamper the clinical use of these natural compounds in the normal aging process, as there are few clinical studies in this setting, and polyphenols are generally reported to have low bioavailability, particularly upon oral administration. Moreover, risk assessment and safety evaluation studies to determine the undesirable effects of polyphenols should also be conducted. Consequently, further human clinical trials on individual polyphenols and their combinations should be conducted to clarify the specific roles of these compounds as anti-aging brain molecules.

Importantly, whether resveratrol acts as a direct SIRT1 activator remains debated, as several studies suggest that its biological effects may occur indirectly through modulation of upstream metabolic regulators such as AMPK or through alterations in cellular NAD⁺ metabolism.

#### Metformin

Metformin is an antidiabetic drug widely used to manage type 2 diabetes (T2D) by enhancing insulin sensitivity (Al-Kuraishy et al., 2018). Metformin has pleiotropic effects, including anti-inflammatory, antioxidant, and anti-apoptotic effects, mediated by modulation of various cellular signaling pathways (Alrouji et al., 2024). Many reports highlighted that metformin has an anti-aging effect by reducing blood glucose and hyperinsulinemia, which are implicated in aging (Soukas et al., 2019). Furthermore, metformin reduces age-related cognitive impairment by regulating neuronal autophagy and hippocampal mTOR signaling in the animal model study (Madhu et al., 2022). Metformin preserves brain structure in non-demented T2D patients compared to patients with mild cognitive impairment (Nabizadeh et al., 2022). Metformin enhances neuronal interconnectivity by augmenting synaptic neurotransmission and reducing the accumulation of oxidized macromolecules through induction of neuronal autophagy (Kulkarni et al., 2020). Metformin’s impact on biological aging is primarily attributed to its ability to activate AMPK and SIRT1 while inhibiting mTOR signaling. By modulating the nutrient-sensing pathway that regulates nutrient uptake, signaling, and metabolic processes that drive growth, metformin exerts life-extending effects. A central element of this pathway is the insulin/IGF-1 signaling axis, composed of the insulin/IGF-1 receptor and its substrate IRS2. By suppressing IGF and IRS2 activity, metformin extends lifespan; similarly, mice with reduced IGF receptor or IRS2 expression exhibit increased longevity. Beyond this, metformin supports DNA repair, stimulates mitochondrial biogenesis, and enhances autophagy (Hsu et al., 2021; Kulkarni et al., 2020). Studies have also shown that metformin upregulates SIRT1 expression in the brain, thereby offering neuroprotection against conditions such as subarachnoid hemorrhage. In addition, it activates the unfolded protein response (UPR) to reduce endoplasmic reticulum stress by repressing miR-132 (Docrat et al., 2020). Additionally, metformin was found to elevate SIRT1 and the mitochondrial biogenesis marker PGC-1α by repressing miR-148a. Thus, epigenetic regulation of mitochondrial biogenesis maintenance by metformin in diabetic C57BL/6 mouse whole brain tissue (Docrat et al., 2020) suggests that, beyond its anti-hyperglycemic role, metformin mediates neuroprotection through epigenomic and integrated stress responses in diabetic mice. In addition, metformin has a neuroprotective role against neurodegenerative diseases such as AD and PD by increasing the expression of the brain SIRT1 mRNA (Alrouji et al., 2023). A preclinical study showed that metformin primarily improves oxidative stress and SIRT1 expression in accelerated aging compared with natural aging in rats (Garg et al., 2017). Metformin supplementation has been shown to suppress d-galactose–induced increases in SIRT1, IL-6, and TNF-α, while enhancing Beclin-1 expression (Garg et al., 2017). This indicates that metformin helps restore antioxidant balance and promotes healthy brain aging by stimulating autophagy and reducing inflammation. Moreover, metformin counteracts cellular senescence by downregulating p16, p21, and other SASP mediators. It also elevates DICER1 levels in both mice and humans through a post-transcriptional mechanism involving the RNA-binding protein AUF1. Metformin alters AUF1’s subcellular distribution, disrupting its binding to DICER1 mRNA and stabilizing the transcript, thereby enabling DICER1 accumulation (Noren Hooten et al., 2016). Thus, metformin restores antioxidant status and promotes healthy brain aging by activating autophagy and reducing inflammation.

In addition, metformin delays neuronal senescence and maintains neuronal function by modulating SASP release (Kulkarni et al., 2020). It also enhances nutrient sensing, boosts autophagy, improves intercellular communication, protects against macromolecular damage, slows stem-cell aging, regulates mitochondrial activity and transcription, and reduces telomere shortening and senescence (Kulkarni et al., 2020). Metformin also exerts several AMPK-independent actions, including direct activation of SIRT1, inhibition of mTORC1 through Rag-GTPases, suppression of adipogenesis via the p70S6K pathway, induction of DNA damage-like signaling through ATM/Chk2 activation, and stimulation of Nrf2, collectively leading to reduced inflammatory responses. Its role in maintaining energy balance and body weight has been linked to growth/differentiation factor 15 (GDF15), underscoring the need to investigate further metformin’s GDF15-mediated regulation of biological aging (Coll et al., 2020). These properties position metformin as a promising gerotherapeutic candidate suitable for clinical translation. In addition, metformin demonstrates anti-senescence effects on neurons (Acar et al., 2021). It regulates brain cellular senescence by activating AMPK and enhancing the expression of the neuroprotective factor BDNF (Ameen et al., 2022). Therefore, metformin helps prevent brain atrophy during aging. These verdicts highlighted that metformin, by activating brain SIRT1, attenuates brain aging and related neurodegenerative diseases. However, there are some limitations to the administration of metformin in the clinic due to its short half-life and relatively low bioavailability. The design of novel drug delivery systems to improve metformin bioavailability, enhance its stability, and reduce its side effects is widely appreciated in clinical applications. Lastly, the distinct molecular mechanisms by which metformin attenuates aging and various aging-related diseases are still poorly understood and warrant further investigation (Zhang et al., 2025). A good understanding of these mechanisms will momentously facilitate the development of novel and efficient strategies for the prevention and treatment of aging and aging-related diseases with metformin.

#### Statins

Statins are cholesterol-lowering drugs commonly used to treat dyslipidemia, acting by inhibiting HMG-CoA reductase, a rate-limiting enzyme for *denovo* cholesterol biosynthesis (Ameen et al., 2022). In addition, statins have potent antioxidant, anti-inflammatory, and anti-atherogenic effects (Kadhim et al., 2020). Statins have conflicting effects on neurodegenerative diseases (Al-Kuraishy et al., 2024). Many studies showed that statins promote the expression of the anti-aging gene *klotho* in cyclosporine-induced nephropathy in mice. In addition, by inhibiting the RhoA pathway, statins promote klotho gene expression in Mimcd3 cells, suggesting an anti-aging effect of statins on vascular aging. Statins are neuroprotective against aging-related neurodegenerative diseases such as AD and PD (Samaras et al., 2019). Statins improve brain function through a cholesterol-independent mechanism (Fracassi et al., 2019). Of interest, atorvastatin improves high-fat diet-induced cognitive impairment in mice by activating the brain SIRT1 signaling pathway. Mounting evidence has demonstrated that diet-induced obesity is associated with cognitive impairment via increasing oxidative stress and inflammation in the brain. Notably, moderate doses of atorvastatin were significantly greater than the low dose of atorvastatin in improving brain function by SIRT1 activation (Liu et al., 2019). Carloni et al. (Carloni & Balduini, 2020) observed that simvastatin attenuates hypoxic-ischemic brain injury in rats by activating the brain SIRT1 signaling pathway.

Moreover, simvastatin potentiates the autophagy response induced by neonatal hypoxia-ischemia, as shown by the increased expression of LC3 and BECLIN 1, increased monodansylcadaverine, and reduced expression of p62. The autophagy inhibitor 3-methyladenine (3MA) completely blocked simvastatin’s neuroprotective effect. Simvastatin preconditioning also prevented the hypoxia-ischemia-induced depletion of SIRT1, an effect completely blocked by 3MA (Carloni & Balduini, 2020). These findings suggest that simvastatin preconditioning may modulate autophagy and survival pathways by affecting mTORC1, mTORC2, and SIRT1 activities. Furthermore, statins have neuroprotective effects against aging and age-related diseases by inhibiting neuronal senescence in patients with various neurodegenerative diseases (Torrandell-Haro et al., 2020). Remarkably, statins have potential senomorphic effects against neuronal cellular senescence (Zhang et al., 2023). Simvastatin inhibits SASP release by blocking protein prenylation and regulating Rho-GTPase (Liu et al., 2015). Therefore, statins that activate SIRT1 and modulate inflammatory and oxidative stress may reduce brain aging.

Nevertheless, numerous limitations inherent to observational studies may lead to bias or misclassification of neuroimaging brain measures in older subjects treated with statins. Despite adjustment for common confounders, statins may also be related to the risk of dementia. Another limitation is that statin dosage was not recorded and, therefore, not assessed. Lipophilic statins such as atorvastatin and simvastatin are more likely to cross the BBB and penetrate the brain compared to hydrophilic statins such as pravastatin (Ameen et al., 2022). Thus, the generalizability of the neuroprotective effects against brain aging and associated neurological disorders needs further study.

### Non-Pharmacological SIRT1 Activators

#### Caloric Restrictions

Dietary interventions such as caloric restriction prolong lifespan and health span by slowing the aging process, benefiting general health, and improving memory performance. Caloric restriction also retards the progression of diverse age-related diseases, such as AD. However, the specific molecular basis of these effects remains unclear. A better understanding of the pathways underlying these effects could pave the way to novel preventive or therapeutic strategies (Van Cauwenberghe et al., 2016). Caloric restriction promotes healthy brain aging by modulating key molecular targets, including Insulin/IGF-1, mTOR, and SIRTs. However, caloric restriction requires a strict regimen and can be challenging to maintain (Trisal & Singh, 2024). A potential alternative to caloric restriction as a lifestyle modification is the use of caloric restriction mimetics. These compounds mimic the biochemical and functional effects of caloric restriction without requiring a reduction in energy intake (Trisal & Singh, 2024). Caloric restriction mimetics, such as rapamycin, metformin, Resveratrol, spermidine, and others, have emerged as promising anti-aging molecules that mimic the beneficial effects of caloric restriction (Trisal & Singh, 2024; Van Cauwenberghe et al., 2016). Findings from a preclinical study demonstrated that caloric restriction improves cognitive function by increasing hippocampal expression of SIRT1 and BDNF in mice (Wei et al., 2023). Atalay et al. found that intermittent or continuous caloric restriction enhances hippocampal SIRT1 expression in a neurodegenerative mouse model (Atalay et al., 2025).

Furthermore, caloric restriction mimetics enhance antioxidant defense systems by activating the Nrf2 pathway, inhibit ROS generation by attenuating mitochondrial dysfunction, and regulate redox-sensitive signaling pathways, such as the PI3K/Akt and MAPK pathways, thereby promoting neuronal cell survival and preventing unhealthy brain aging (Sharma & Singh, 2023). Caloric restriction has also been recognized for its role in counteracting chronic inflammation associated with cancer during aging. Consistently, calorie restriction mimetics increase SIRT1 levels and function as autophagy activators, with potential implications for cancer prevention (Kim et al., 2015). These findings highlighted that caloric restriction and caloric restriction mimetics, by regulating SIRT1 and other signaling pathways, can attenuate cognitive impairment and neurodegeneration during brain aging.

#### Physical Exercise

Mounting evidence suggests that physical exercise is the most effective non-pharmacological strategy to advance brain health (Moradikor, 2025). Physical exercise prevents cognitive decline associated with aging and reduces the risk of developing neurodegenerative diseases. The positive effects of physical exercise can be attributed to increased neurogenesis and neuroplasticity, leading to improvements in learning and memory. At the molecular level, there is a strong consensus that BDNF is the crucial molecule underlying the positive effects of physical exercise on the brain (Cefis et al., 2023). Although physical exercise undeniably leads to beneficial effects through BDNF expression, the cellular sources and molecular mechanisms underlying physical exercise-induced cerebral BDNF overproduction are still being elucidated (Cefis et al., 2023). The positive effects of physical exercise on the brain may be explained by increased hippocampal neurogenesis, enhanced long-term potentiation, and regulation of synaptic plasticity.

Furthermore, physical exercise has beneficial effects against aging-related changes, dopaminergic neuron vulnerability, and PD progression by significantly increasing SIRT1 levels and activating other neuroprotective pathways in rats. The exercise-induced increase in SIRT1 may mediate the effects of exercise on the nigral renin-angiotensin system (RAS). However, exercise may increase VEGF levels and modulate RAS activity via distinct pathways. Physical exercise, via the RAS, contributes to the inhibition of a pro-oxidative and pro-inflammatory state that increases dopaminergic neuron vulnerability and the risk of PD with aging (Muñoz et al., 2018). It is recognized that physical exercise promotes learning and memory formation and alleviates the symptoms of depression by inducing *Bdnf* gene expression and signaling in the hippocampus. Remarkably, lactate produced during physical exercise crosses the BBB and promotes hippocampal-dependent learning and memory in a BDNF-and SIRT1 SIRT1-dependent manner (El Hayek et al., 2019). Henceforth, physical exercise may inhibit brain aging-induced cognitive impairment and associated neurodegeneration, mainly by activating the brain SIRT1 signaling pathway.

Overall, SIRT1 activators have a neuroprotective effect against brain aging by regulating inflammatory and oxidative stress disorders through modulation of different signaling pathways [Table 4**]**. Thus, SIRT1 activators can prevent the development and progression of cognitive impairment and dementia in elderly subjects.

**Table 4:** Role of SIRT1 activators in brain aging

SIRT1 activators	Findings	Ref.
SRT1720	SIRT1 activator SRT1720 inhibits the production of inflammatory cytokines and cellular senescence both in humans and animals.	(Sung et al., 2021).
Resveratrol	Resveratrol is effective against brain aging by activating SIRT1. However, at higher concentrations, Resveratrol induces apoptosis of senescent neurons. Therefore, Resveratrol has dual senolytic and senomorphic effects on senescent neurons in the aging brain by activating the SIRT1 signaling pathway.	(Mazucanti et al., 2015). (Zhang et al., 2023).
Metformin	Metformin activates AMPK and SIRT1 while inhibiting mTOR signaling. Metformin supports DNA repair, stimulates mitochondrial biogenesis, and enhances autophagy. Metformin delays neuronal senescence and maintains neuronal function by modulating SASP release. Metformin also exerts several AMPK-independent actions, including direct activation of SIRT1, inhibition of mTORC1 through Rag-GTPases, suppression of adipogenesis via the p70S6K pathway, induction of DNA damage-like signaling through ATM/Chk2 activation, and stimulation of Nrf2, collectively leading to reduced inflammatory responses. Metformin has anti-senescence effects on neurons and regulates brain cellular senescence by activating AMPK and enhancing the expression of the neuroprotective factor BDNF.	(Hsu et al., 2021; Kulkarni et al., 2020). (Kulkarni et al., 2020), (Coll et al., 2020). (Acar et al., 2021), (Ameen et al., 2022).
Statins	Atorvastatin improves high-fat diet-induced cognitive impairment in mice by activating the brain SIRT1 signaling pathway. Simvastatin attenuates hypoxic-ischemic brain injury in rats by activating the brain SIRT1 signaling pathway. Statins have neuroprotective effects against aging and aging-related diseases by inhibiting neuronal cellular senescence in patients with different neurodegenerative diseases. Statins have potential senolytic effects against neuronal cellular senescence.	(Liu et al., 2019). (Carloni & Balduini, 2020). (Torrandell-Haro et al., 2020). (Zhang et al., 2023).
Caloric restriction	Caloric restriction prolongs lifespan and healthspan by slowing the aging process, improving overall health, and enhancing memory performance. Caloric restriction promotes healthy brain aging by modulating key molecular targets, including insulin/IGF-1, mTOR, and SIRTs. Caloric restriction mimetics, such as rapamycin, metformin, Resveratrol, and spermidine, have anti-aging effects. Caloric restriction improves cognitive function by increasing hippocampal expression of SIRT1 and BDNF in mice. Intermittent or continuous caloric restriction enhances hippocampal SIRT1 expression in a neurodegenerative mouse model. Caloric restriction mimetics that enhance antioxidant defense systems promote neuronal cell survival and prevent unhealthy brain aging. Caloric restriction counteracts chronic inflammation associated with cancer during aging by enhancing SIRT1 levels.	(Van Cauwenberghe et al., 2016). (Trisal & Singh, 2024). (Trisal & Singh, 2024; Van Cauwenberghe et al., 2016). (Wei et al., 2023). (Atalay et al., 2025). (Sharma & Singh, 2023). (Kim et al., 2015).
Physical exercise	Physical exercise is the most effective non-pharmacological strategy to advance brain health. Physical exercise increases hippocampal neurogenesis, enhances long-term potentiation, and regulates synaptic plasticity by increasing SIRT1 and other neuroprotective pathways in rats. Physical exercise promotes learning and memory formation and alleviates the symptoms of depression by inducing *Bdnf* gene expression and signaling in the hippocampus.	(Moradikor, 2025). (Muñoz et al., 2018). (El Hayek et al., 2019).

## Limitations, Knowledge Gaps, and Future Directions

Although increasing evidence supports SIRT1 as a major regulator of aging-associated pathways, several limitations should be considered when interpreting its relevance as a therapeutic target in brain aging (Guarente, 2011; Imai & Guarente, 2014). A large proportion of available data originates from preclinical studies using heterogeneous models, variable intervention timing, and different outcome measures, which may not fully capture the slow and multifactorial nature of physiological brain aging in humans (Kennedy et al., 2014). In addition, SIRT1 functions in a highly context-dependent manner, and its effects may differ across brain regions and between neurons and glial populations, complicating the identification of uniform mechanisms and therapeutic endpoints (Herskovits & Guarente, 2014). From a translational perspective, pharmacological modulation of SIRT1 faces practical challenges, including limited bioavailability, uncertain brain penetration of some compounds, potential off-target effects, and difficulties in defining optimal dosing and treatment duration (Baur & Sinclair, 2006). Moreover, since aging is not a pathological condition per se, the aim of SIRT1 modulation should be framed as supporting neuronal resilience, preserving homeostasis, and delaying functional decline, rather than treating aging itself as a disease. A key knowledge gap remains the identification of reliable biomarkers of CNS target engagement to monitor SIRT1 modulation and predict neuroprotective benefit (Rajman et al., 2018).

Despite promising preclinical evidence, the translational potential of SIRT1 activators in brain aging remains uncertain. Most available data derive from in vitro and animal studies, whereas clinical evidence in humans remains limited and heterogeneous. In addition, pharmacokinetic challenges, including low oral bioavailability, rapid metabolism, and variable blood–brain barrier penetration, may restrict the therapeutic effectiveness of several candidate compounds such as resveratrol. Furthermore, the mechanism by which some polyphenols modulate SIRT1 activity remains debated, as some studies suggest that their effects may occur indirectly through upstream metabolic regulators such as AMPK or by modulating cellular NAD⁺ metabolism rather than through direct activation of SIRT1.

Finally, while multiple SIRT1-modulating strategies have shown promising outcomes in preclinical settings, clinical evidence supporting clear benefits for brain aging phenotypes remains limited and heterogeneous. Future studies should prioritize aged and clinically relevant models, incorporate standardized cognitive and neurobiological endpoints, and clarify therapeutic windows and cell-type specificity. Well-designed clinical trials integrating cognitive measures with molecular and imaging biomarkers will be essential to determine whether SIRT1-targeting interventions can be translated into meaningful benefits for age-associated cognitive decline and neurodegenerative vulnerability (Rajman et al., 2018).

## Conclusions

Aging is a multifactorial process that affects various tissues, including the brain. SIRT1 is necessary for brain development and control of brain senescence by promoting axonal growth, dendritic branching, and neurite outgrowth. In addition, SIRT1 enhances memory function by activating synaptic plasticity and preventing age-related neurodegeneration and cognitive decline. SIRT1 has an anti-aging effect by deacetylating different proteins involved in cellular pathways, such as apoptosis and stress response. Therefore, SIRT1 has neuroprotective effects against the development and progression of brain aging and related neurodegenerative diseases. Notably, SIRT1 is highly dysregulated in the aging brain and other aging processes. Therefore, SIRT1 activators may be a promising therapeutic strategy against brain aging. SIRT1 activators, such as Resveratrol, metformin, and statins, have neuroprotective effects against brain aging by regulating inflammatory and oxidative stress pathways through modulation of downstream signaling pathways. Additional preclinical and clinical studies are warranted in this regard. The more thorough clinical study is therefore suggested to elucidate these findings.

## Data Availability

This manuscript does not report data generation or analysis.
